# Author Correction: Thymoquinone therapy remediates elevated brain tissue inflammatory mediators induced by chronic administration of food preservatives

**DOI:** 10.1038/s41598-019-50175-3

**Published:** 2019-11-13

**Authors:** Ahmed Mohsen Hamdan, Mohammed M. Al-Gayyar, Mohamed E. E. Shams, Udai Salamh Alshaman, Kousalya Prabahar, Alaa Bagalagel, Reem Diri, Ahmad O. Noor, Diena Almasri

**Affiliations:** 1 Department of Pharmacy Practice, Faculty of Pharmacy, University of Tabuk, Tabuk, Saudi Arabia; 2 Department of Pharmaceutical Chemistry, Faculty of Pharmacy, University of Tabuk, Tabuk, Saudi Arabia; 3 Department of Biochemistry, Faculty of Pharmacy, Mansoura University, Mansoura, Egypt; 4 Department of Pharmaceutics, Faculty of Pharmacy, Mansoura University, Mansoura, Egypt; 5 Department of Pharmaceutics and Pharmacy Practice, Oman College of Health Sciences, Pharmacy Program, Ministry of Health, Muscat, Oman; 6 Department of Pharmacy Practice, Faculty of Pharmacy, Kind Abdulaziz University, Jeddah, Saudi Arabia

Correction to: *Scientific Reports* 10.1038/s41598-019-43568-x, published online 07 May 2019

The original version of this Article contained an error in Affiliation 6, which was incorrectly given as ‘Department of Clinical Pharmacy, Faculty of Pharmacy, Kind Abdulaziz University, Jeddah, Saudi Arabia’. The correct affiliation is listed below:

Department of Pharmacy Practice, Faculty of Pharmacy, Kind Abdulaziz University, Jeddah, Saudi Arabia.

This error has now been corrected in the HTML and PDF versions of the Article, and in the accompanying Supplemental Material.

